# Baseline diet quality and adequacy of AU‐ARROW participants‐ Preliminary findings

**DOI:** 10.1002/alz70860_106476

**Published:** 2025-12-23

**Authors:** Malika G Fernando, Juliana Chen, Carolina B Castro, Stephanie Jane Fuller, Samantha L Gardener, Sharon L. Naismith, Laura D Baker, Miia Kivipelto, Victor L. Villemagne, Stuart Grieve, Paul A Yates, Nicola Armstrong, Kaarin J. Anstey, Hamid R Sohrabi, Manohar Garg, Ralph N Martins

**Affiliations:** ^1^ Macquarie University, Sydney, NSW, Australia; ^2^ The University of Sydney, Sydney, NSW, Australia; ^3^ Murdoch University, Murdoch, Western Australia, Australia; ^4^ Alzheimer's Research Australia, Perth, Western Australia, Australia; ^5^ Edith Cowan University, Perth, Western Australia, Australia; ^6^ Wake Forest University, Winston‐Salem, NC, USA; ^7^ University of Eastern Finland, Kuopio, Finland; ^8^ Karolinska Institutet, Stockholm, Sweden; ^9^ University of Melbourne, Melbourne, VIC, Australia; ^10^ Austin Health, Heidelberg, VIC, Australia; ^11^ The University of Pittsburgh, Pittsburgh, PA, USA; ^12^ Curtin University, Perth, Western Australia, Australia; ^13^ University of New South Wales, Sydney, NSW, Australia

## Abstract

**Background:**

Recent studies indicate that lifestyle interventions combining a healthy diet, physical activity, brain training and health monitoring are effective in preventing dementia and related cognitive decline. This preliminary analysis aimed to assess the baseline dietary adequacy and quality of participants of The Australian‐Multidomain Lifestyle Intervention to reduce dementia risk (AU‐ARROW) which is an ongoing 2‐year randomized clinical trial.

**Method:**

Data from 247 cognitively unimpaired adults enrolled in AU‐ARROW were included (68% female; mean age 67±5 years). Nutrient intake and dietary adequacy at baseline were assessed through data from a self‐administered validated Cancer Council of Victoria Food Frequency Questionnaire. Dietary quality was assessed by comparing dietary intake against five food groups and discretionary foods in the Australian Dietary Guidelines (2013).

**Result:**

Participants’ average energy intakes were 8057±2054kJ/day (females) and 8872±2060kJ/day (males) and the macronutrient contributions to energy were 40% carbohydrate, 39% fat and 21% protein, indicating higher fat and lower carbohydrate intakes compared to acceptable macronutrient distribution ranges. Total fat intake comprised 34% saturated fat, 47% monounsaturated fat and 19% polyunsaturated fat, exceeding recommended levels for saturated fat. Daily consumption of five food groups by females and males averaged 3.6 and 3.2 serves of vegetables, pulses and beans, 1.3 and 1.6 serves of fruits, 3.9 and 4.7 serves of grains/cereals foods, 3.1 and 3.2 serves of meat, poultry, fish, eggs and nuts and 1.7 serves of milk, yoghurt, cheese and/or alternatives, respectively. Discretionary food intake averaged 4.2 serves for females and 4.6 serves for males (Females:1.9 cream and butter,1.8 sweets and confectionary food, 0.3 processed‐meat, 0.2 fast‐foods; Males:2 cream and butter, 2 sweets and confectionary food, 0.4 processed‐meat, 0.2 fast‐foods). Average intake of unsaturated spreads and oils were 24.6±19.6g/day (female) and 28.2±20.0g/day (male).

**Conclusion:**

Baseline nutrient adequacy and diet quality of AU‐ARROW participants were poor, characterized by insufficient intakes of vegetables, pulses and beans, fruits and dairy foods, but excessive intake of discretionary food, likely contributing to higher saturated fat intakes. These findings align with the diet quality of the broader Australian population. The dietary intervention of AU‐ARROW aims to address these nutrient imbalances and improve overall diet quality.

